# Hepatitis B Associated Monoclonal Gammopathy That Resolved after Successful Liver Transplant

**DOI:** 10.1155/2009/103784

**Published:** 2009-02-24

**Authors:** P. Sreenivasan, S. Nair

**Affiliations:** Section of Hepatology, Division of Gastroenterology, Department of Medicine, Methodist University Hospital, University of Tennessee Health Science Center, Memphis, TN 38104, USA

## Abstract

Monoclonal gammopathy of undetermined significance (MGUS) has been most commonly associated with diseases like multiple myeloma, Waldenstrom's macroglobulinemia, primary systemic amyloidosis, HIV, and other lymphoproliferative disorders. There has been an isolated report of MGUS in patients coinfected with HIV and Hepatitis B, as the work by Amara et al. in 2006. Here, we report a case of IgA-kappa light chain gammopathy secondary to Hepatitis B infection, which resolved after liver transplantation. To our knowledge, this is the first reported case of M protein spike seen in the context of Hepatitis B infection only.

## 1. Case

Mr. SD, a 45-year-old Caucasian male, was referred for evaluation for liver transplantation because of end-stage liver disease caused by Hepatitis B. He had been well until six months prior to his initial visit, when he started losing weight and developed severe fatigue. During the six-month period, he had lost 25 kilograms.

His past medical history was significant for non-Hodgkin's lymphoma that was diagnosed eleven years ago. He was treated with 8 cycles of CHOP chemotherapy and had been in remission since then.

On examination, the patient appeared mildly icteric. The abdomen was soft with some tenderness in the right upper quadrant and ascites. The spleen was mildly enlarged. The remainder of the examination was normal. No enlarged lymph nodes were detected.

Complete blood count showed a leukocyte count of 2500 cells/mm^3^, hemoglobin of 9.8 gm/dL, and platelet count of 29 000 cells/mm^3^. His liver function tests were abnormal with a total bilirubin of 5 mg/dL (normal, 0.2–1.2 mg/dL), ALT: 361 U/L (normal, 17–63 U/L), AST: 750 IU/L (normal, 15–40 U/L), total protein: 15.4 g/dL (normal, 6.1–7.9 g/dL), albumin: 2.9 g/dL (normal, 3.9–4.8 g/dL), and an INR of 1.9. Serological tests for viral hepatitis were as follows: Hepatitis B surface antigen positive (HbsAg), Hepatitis B surface antibody negative (antiHBs), HBe antigen negative, HBe antibody positive, IgM antibody to Hepatitis B core antigen positive, IgG antibody to Hepatitis B core antigen positive, and HBV DNA 4 150 000 IU/mL (by PCR). Antibodies against hepatitis delta virus and hepatitis A virus were negative. HCV antibody was negative. Hepatitis C RNA was undetectable. Cytomegalovirus IgG and IgM were negative. Ebstein Barr virus IgM was negative and IgG was positive. He had a negative antinuclear antibody and a negative antismooth muscle antibody. HIV antibody was negative. Esophagogastroduodenoscopy revealed mild esophagitis and erythema of the gastric body. The duodenal biopsies were normal. Colonoscopy was normal. CT scan and ultrasound examination of the abdomen showed a small liver with moderate ascites, splenomegaly, patent portal, and hepatic veins. There were no enlarged lymph nodes on CT scan. Liver biopsy confirmed cirrhosis from HBV.

**Figure 1:**
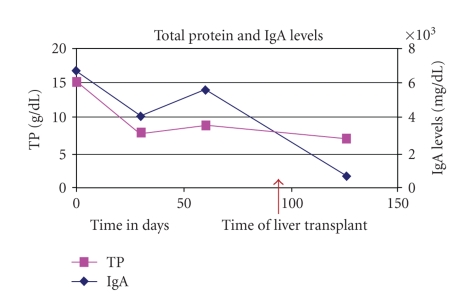
Total protein and IgA levels.

The striking elevation in his globulin fraction (12.5 g/dL) was further worked up. Serum protein electrophoresis showed the presence of a large monoclonal protein spike (M protein) in the gamma region which on immunofixation electrophoresis (IFE) was identified to be IgA-kappa type (see Figure 1). The IgA levels were increased at 6730 mg/dL (reference range 70–400 mg/dL) and the IgG and IgM levels were decreased at 466 mg/dL (reference range 700–1600 mg/dL) and 25.6 mg/dl (reference range 40–320 mg/dL), respectively. Urine was negative for M protein. Cryoglobulins were negative. Serum viscosity was 3.3 (normal, 1.4–1.8). A bone marrow biopsy on two separate occasions showed normocellular bone marrow with less than 2 percent population of plasma cells. The plasma cells were predominantly kappa light chains on the in situ hybridization. Bone marrow cytogenetics did not detect any abnormalities. These results were reproduced on 2 repeat bone marrow biopsies. Metastatic bone survey showed no myelomatous lesions. Full body positron emission tomography (PET) scan and CT scan were performed, and no focus suspicious for lymphoma or myeloma or infection was identified. Whole body MRI was unremarkable. On retrospective review, the patient had an elevated total protein (12 g/dL) approximately six months prior to initial visit.

Within the next few weeks, the patient continued to deteriorate, and he developed worsening ascites with hyponatremia. It was clear that the patient had progressive liver disease from chronic Hepatitis B with a relatively high MELD score of 23, and he would benefit from a liver transplantation. Our dilemma before proceeding with liver transplantation was whether he had an underlying hematological malignancy that was causing the monoclonal gammopathy. This was even more relevant given his prior history of lymphoma. We were also concerned about potential vascular complications after liver transplant given his increased viscosity. However, due to his deterioration and absence of any clear-cut evidence of malignancy, it was decided to place the patient on the liver transplant wait list. We chose not to anticoagulate him since he already had an elevated INR.

Anti-HBV therapy was initiated with a combination of entecavir (0.5 mg/day) and tenofovir (300 mg/day) to decrease his HBV DNA to the lowest possible level before transplantation. However, he was on this antiviral therapy for less than a month as he received a deceased donor liver transplant ten days after listing. In the period between listing and liver transplantation, his bilirubin continued to trend up from 3.3 mg/dL to 4.6 mg/dL, and his total protein level fluctuated between 12.5 g/dL and 13.5 g/dL.

Six weeks after his liver transplant, his HBV DNA level was undetectable. He cleared his Hepatitis B surface antigen four months after transplant. His IgA levels normalized. As per standard protocol in our institution, he received two doses of rabbit antithymocyte globulin at 1.5 mg/kg during the time of transplant and on postoperative day 2. He also received tacrolimus and mycophenolate mofetil for 3 months. Subsequently, the mycophenolate mofetil was withdrawn, and he is currently on tacrolimus monotherapy with trough levels between 3–5 mg/mL. He continues to receive Hepatitis B immunoglobulin infusion every month along with entecavir (0.5 mg/day), and his Hepatitis B antibody titre has been kept above 300 mIU/mL. He is currently 9 months posttransplant, his total protein levels range 4-5 g/Dl, and he has had no detectable M protein spike.

The temporal relation of the liver transplant with disappearance of Hepatitis B surface antigen and normalization of the monoclonal spike implicates Hepatitis B as the possible cause of the M protein spike.

## 2. Discussion

There is very little data and uncertainty regarding the incidence and natural history of M proteins in a person with Hepatitis B. It has been suggested that the immunological response elicited against Hepatitis B virus in the host rather than the direct cytopathic effect of the virus may be the basis for the pathogenesis of hepatic and nonhepatic manifestations [2]. Hepatitis B virus seems particularly well suited to initiate a chronic immune disease because of its tendency to persist in spite of a good immune response. This may cause clonal expansion of the immunoglobulin secreting cells and may explain the above phenomenon. Alternately, the M protein spike could represent increased serum immunoconglutinin titres. It has been previously suggested that elevated serum immunoconglutinin titers in patients with acute and chronic hepatitis B virus infection may represent a physiological autoimmune response to HbsAg/anti-HBs immune complexes [3]. Although our patient did not have the anti-HBs antibody, it has been suggested that in the light of massive antigenemia present in chronic HbsAg carriers, it is possible that anti-HBs exists in the form of HbsAg/anti-HBs immune complexes. This would make it difficult to detect anti-HBs in serum samples by conventional serological methods [4].

There were several intriguing aspects to this patient. The primary issue was whether he was eligible for liver transplantation in the presence of an increasing M protein. The fact that he had lymphoma 10 years ago complicated this evaluation. Additionally, an association between Hepatitis B infection and non-Hodgkin's lymphoma has been previously described [5]. However, he had had regular follow-ups every six months after remission of lymphoma, and also his malignancy workup prior to transplant was negative. Moreover, the M protein spike was noticed only 6 months prior to transplant and this coincided with the elevation of his liver function tests. Surprisingly, his IgM antibody to Hepatitis B core antigen was positive, as was his total core antibody. He did have active hepatitis and this can be deduced by the fact that he had an ALT level that was 10 times the upper limits of normal; his explanted liver showed significant inflammatory activity. His biopsy also revealed frank cirrhosis. By putting all this together, it was felt that most likely he had a flare of his chronic HBV, which might have triggered the elevation in M protein.

Because of his elevated M protein and an urgent need for liver transplantation, we decided to initiate dual therapy with a nucleoside (entecavir) and nucleotide (tenofovir) analogue to decrease his HBV DNA. However, he was on therapy less than a month before a suitable organ became available. We decided to accept the organ because of high MELD score rather than to wait for HBV DNA disappearance. Since we were planning to continue therapy posttransplant along with HBIG (target anti-HBs titers >500 IU/mL first three months and levels of >300 IU/mL after three months), we believed that the risk of recurrence of HBV was very small. Unfortunately in the time period before the transplant and after initiation of antiviral therapy, we failed to follow his IgA levels and HBV DNA levels. However, his protein levels remained unchanged suggesting that there may have been very little change in his gammopathy, and this is not surprising given the fact that nucleos(t)ide therapy seldom leads to HBsAg clearance in that time frame.

After liver transplantation and with clearance of the Hepatitis B surface antigen, the M protein levels became normal. There are two potential explanations for the disappearance of the M protein spike. One and the most likely reason is the removal of antigen source with native hepatectomy. His Hepatitis B surface antigen, M protein, and total protein levels remain within normal limits 1 year after transplantation. The second possibility is the impact of thymocyte, given at the time of transplant, on plasma cells. Antithymocyte globulin has been used in treatment of plasma cell dyscrasias [6] due to its activity against several plasma cell antigens. This is less likely in our patient because thymocyte globulin was used only as induction therapy at the time of transplantation. The elimination half time of thymoglobulin is 2-3 days and is unlikely that its effect on plasma cell is sustained for a year [6]. In conclusion, this case demonstrates that monoclonal gammopathy can be associated with chronic Hepatitis B infection and can be eliminated after successful transplantation.
